# Health care provider's risk perception, and preparedness towards COVID-19 pandemic in North Central Ethiopia, 2020

**DOI:** 10.1016/j.heliyon.2021.e06610

**Published:** 2021-03-29

**Authors:** Binyam Minuye Birihane, Wubet Alebachew Bayih, Yohannes Tesfahun, Tigabu Munye, Abebaw Yeshambel Alemu, Demeke Mesfin Belay

**Affiliations:** College Health Sciences, Debretabor University, Debretabor, Ethiopia

**Keywords:** Risk perception, Coronavirus, Preparedness, Health care provider, Ethiopia

## Abstract

**Background:**

Risk perception, positive emotions, and preparedness are important parameters in predicting pandemic protective behaviors. Though, health care providers are required with sufficient knowledge, skills, preparedness and best practices towards corona virus 2019, there are limited studies in Ethiopia.

**Aim:**

This study aimed to assess health care providers’ level of risk perception, preparedness and its associated factors among HCWs in North Central Ethiopia, 2020.

**Methods:**

An institutional-based cross-sectional survey was conducted among 217 health care providers working in South Gondar zone Hospitals from May 15–30, 2020. Data were collected using a self-administered questionnaire. Data were coded, entered, cleaned and checked using Epi data statistical software version 4.2.0.0 and taken in STATA Version 14 statistical software for analysis. Binary logistic regression was used for the analysis. Odds ratio along with 95% CI were estimated to measure the strength of the association. Descriptive statistics are presented in figures, text, and tables.

**Findings and conclusion:**

The level of high risk perception among frontline health care workers was 57.6% (95% CI: 56.9, 58.3). Almost half, 49.8% health care providers were prepared for corona virus pandemic and only 43.78% of health care providers had good knowledge regarding COVID -19. Having good knowledge (Adjusted Odd Ratio (AOR) = 2.83; 95% CI: 1.49, 5.34), training on COVID -19 (AOR = 2.16; 95% CI:1.07, 4.39), and avoiding meeting suspected/confirmed of COVID -19 (AOR = 2.67; 95% CI:1.05, 6.83) were significantly associated with risk perception. Risk perception, knowledge and preparedness for corona virus pandemic were low. Ensuring the improvement of knowledge, preparedness, and encouragement is important.

## 1 Introduction

The coronavirus disease 2019 (COVID-19) pandemic is a new disease caused by severe acute respiratory syndrome coronavirus 2 (SARS–CO–V-2), which was first reported in Wuhan, Hubei province, China in December 2019 [1,2]. Ethiopia reported its first confirmed case of COVID-19 on March 13, 2020 [3].

Health care workers (HCWs) are at high risk of acquiring COVID- 19 while caring for patients [4]. Risk perception is an abstract and socially constructed phenomenon, with responses to risk events often difficult to predict [5]. Risk perception is influenced by different factors. HCWs not only have to work harder for longer hours; they often do so in a context where the knowledge and understanding of the novel pathogen is still suboptimal [6]. They faced different challenges, such as risk of getting infected of COVID 19, easily transmitted to other patients, families and friends [7], fear,anxiety, stress,depression, emotion due to the pandemicity of COVID 19 [8, 9, 10]. It has also an economic and sociocultural impact on the patient as well as health care providers [11]. Minimizing direct contact with patients and not reporting to work, higher levels of psychological distress [12,13],post-traumatic stress [14], physical damage, skin damage [15], irritable and upset [16] are some of impact of pandemic.

The rapid transmission and burden of COVID 19 requires coordinated action across many areas of the healthcare system staffing, equipment supply chains, nursing and medical treatment and infection control [17], individual behavior such hand hygiene, avoiding public gathering, physical distancing [18,19]. More than 90% of people will adopt one or more preventive behaviors. However, apart from hand washing, less than 50% of the respondents practiced preventive behaviors such as avoiding crowded places or public transport [20].

Positive emotions, perceived control, and risk perception are factors affecting pandemic protective behaviors which affects hand hygiene, personal protective use, and avoidant behaviors [21, 22, 23]. In addition, prompt and proper public health interventions addressing cultural impact and risk for stigmatization along with proper screening, treatment, and follow up for affected individuals and close contacts can reduce the number of infections, serious illness, and deaths. Regular mental health care, health education and counseling for these care providers is very important to combat COVID 19 and its effect [24,25]. Bringing a behavioral change, taking immediate action and providing the right information to help people to adopt effective measures against the spread is important [26].

Knowledge, and attitude towards the pandemic influence preparedness, risk perception, practice of health care providers and willingness to care for COVID-19 patients [27,28], affect preventive behaviors [29]. Preparedness reduce the economic, social, and health related impact of a pandemic [30]. Hence, health care providers should be ready and well prepared with the best available information and protocols to treat any patient with suspected COVID-19 infection.

Although, the concern of COVID-19 causing critical illness and death is at the core of public anxiety; health care provider's preparedness and risk perception towards corona viruses is not addressed in Ethiopia. Therefore, this study was amid in assessing health care workers preparedness, risk perception and associated factors towards COVID-19 in South Gondar Hospitals, North Central Ethiopia, 2020.

## 2 Materials and methods

### 2.1 Study design, period, setting, and population

An institution based cross sectional study was employed in north central Ethiopia, may 14–30/2020. Debre Tabor town is located in South Gondar Administrative Zone of the Amhara regional, state, at a distance of 667 km away from Addis Ababa, the capital city of Ethiopia, 100 km southeast of Gondar and 50 km east of Lake Tana [31]. South Gondar zone has 1 general hospital, 7 primary hospitals, 97 health centers (95 active health centers and 2 treatments centers) and 394 health posts. All Healthcare professionals (HCPs) working in health care institutions (Doctors, nurses, medical lab, pharmacists, anesthetists, midwifery, physiotherapy, and laboratory, public health officer and psychiatrist) working in south Gondar zone hospitals were the source population. A total of 217 HCWs were included during the survey.

### 2.2 Sampling technique

**Figure 1:**
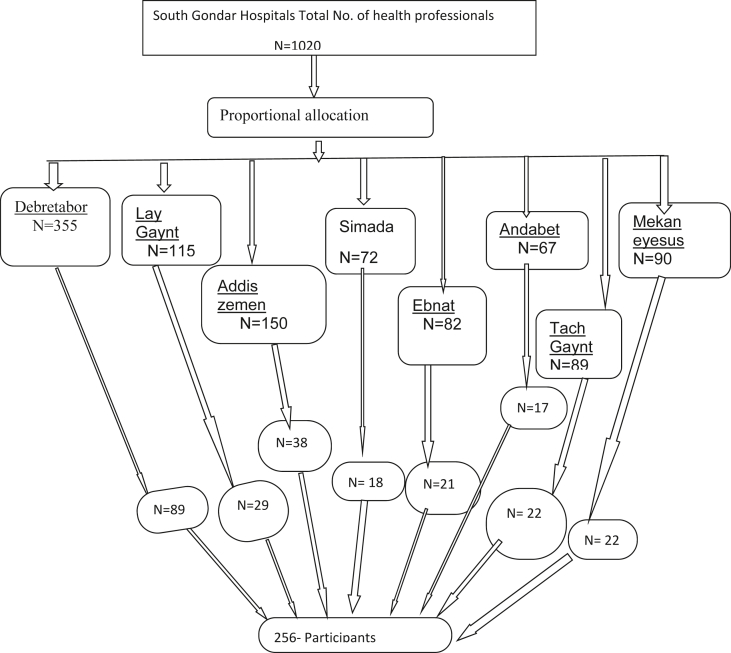
Schematic presentation of sampling procedure.

Health care providers working in 8 hospitals in south Gondar zone (Debre tabor General hospital, Addis Zemen, Anidabet, M/yesus, N/mewuchia, Tachi Gayint, Ebinat and Simada hospitals) were involved. Number of participants from each hospitals was allocated proportionionaly as shown in Figure 1. Health care providers working in south Gondar zone was involved in the study. Number of participants from each hospitals was allocated proportionally as indicated in Figure 1. Since the data collection time was a time when the country was at state of emergency and staff reduction, the study participants were those health care professional found at health care institution during data collection period, selected by simple random technique. All health care providers working in those hospitals were included. HCPs who were ill during data collection period, and annual leave were excluded (Figure 1).

### 2.3 Sample size determination

The sample size (n) required for the study was calculated using the formula to estimate a single population proportion by considering (P) = 81.4% from the study conducted in Ghana [32], Level of significance (α = 5%), Marginal error (D = 5%).

Then n = (Z α_/2_)^2^p (1-P)/(D^2^) n= (1.96)^2^ (0.5 × 0.5) (0.05)^2^

The resulting sample size was 233. By adding non response rate of 10%, the final sample size was 256.

### 2.4 Data collection

Data were collected by using self-administered questionnaire which was adopted from WHO, guidelines issued by National Institute of Health, and previous researches [20,32, 33, 34]. The tool is composed of six parts i.e. Socio-demographic characteristics, knowledge related factors, risk perception related factors, preparedness and preventive measures related factors. The data were collected by four BSc Nurses and one MSC supervisor. Completeness of each recording format was checked before collecting the data. Simple random sampling method was used to select the study participants. Proportional to size allocation method was used to determine the number of respondents from each hospital. Accordingly, participants were selected from eight hospitals.

### 2.5 Variables

#### 2.5.1 Dependent variable

Risk perception (High, Low).

#### 2.5.2 Independent variable

Socio demographic and personal related factors (age, sex, marital status, monthly income, profession, year of experience), chronic illness, information related factors, knowledge, preventive behaviors related factors.

### 2.6 Operational definitions

Knowledgeable–if the score is above or equal to mean score.

Preparedness–if the score is above or equal to mean score.

Risk perception refers to people's judgments and evaluations of hazards they (or their facilities, or environments) are or might be exposed to [35].

High risk perception (health threat)–if the score is above or equal to the mean score.

Low risk perception: if the score is below the mean score.

### 2.7 Data quality control

Pretest was done on 5% of the sample size. Training was given for two consecutive days on how to administer questionnaires, handling ethical issues and maintaining confidentiality and privacy. Completeness of each data collection tools was checked by the principal investigator and the supervisors in a daily base. Double data entry was done by two data clerks and consistency of the entered data was cross-checked.

### 2.8 Data processing and analysis

Data were entered, coded, cleaned and checked by Epi-Data statistical software version 4.2.0.0 and analysis using STATA Version 14 statistical software. Binary logistic regression was used for analysis. Bi-variable analysis was done and all variables that were found to be significant at p-value <0.25 were entered into the multivariable logistic regression model. Independent variables that were significant at p-value <0.05 in the multiple logistic regression models was considered as statistically significant. Finally, the data were presented in texts, figures and tables.

### 2.9 Ethical considerations

Ethical approval letter was obtained from Debre Tabor University, Institutional Health Research Ethics Review Committee (IHRERC). Then Official letter had been written to each health institution for permission and support. The purpose of the study was explained to the study participants, informed written consent was taken. The collected data used for the study purpose.

## 3 Results

### 3.1 Socio demographic factors

The mean age of the respondents was (29.25 ± 5.4) years. Two third, 66.36% of health care providers were males. Majority, 77.88% were in the age group of less than 30 years of age. More than half, 56.48% participants were married. Almost half, 45.62 % were nurses in profession followed by medical doctors, 12.90 %. One hundred fifteen, 53.0% of the participants had monthly income of less than 6682 Ethiopian Birr (ETB) (Table 1).

**Table 1:** Socio demographic characteristics of health care workers working in south Gondar zone, north central Ethiopia.

Socio demographic characteristics	Frequency (%)
Sex	
Male	144 (66.36)
Female	73 (33.64)
Age	
≤30 years	169 (77.88)
>30 years	48 (22.12)
Marital status	
Unmarried	96 (44.44)
Married	120 (55.56)
Profession	
Medical doctor	28 (12.90)
Nurse	99 (45.62)
Medical lab	23 (10.60)
Others	67 (30.88)
Monthly income	
≤6682 ETB	115 (53.00)
>6682 ETB	102 (47.00)
Working experience	
<3 years	45 (20.74)
3–6 years	93 (42.86)
>6 years	79 (36.41)
Any chronic illness	
Yes	23 (10.60)
No	194 (89.40)

Others: pharmacy, midwifery, psychiatry, anesthesia.

### 3.2 Source of information

**Figure 2:**
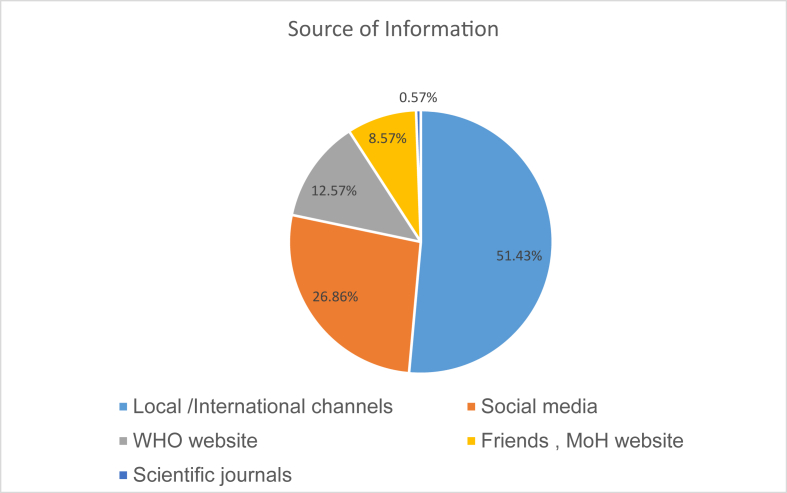
Source of information for health care providers in north central Ethiopia, 2020.

Almost half, 51.43% of health care professional got information from local/international channels, followed by 26.86% from social media. Almost, 91% of health care professional got information daily. Absence/poor internet connection is the main barrier for information access (Figure 2).

### 3.3 Knowledge of health care professional towards COVID 19

**Figure 3:**
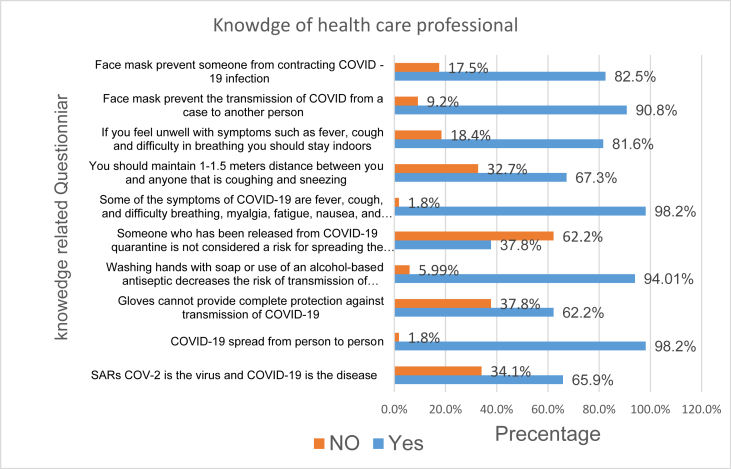
Knowledge of health care providers towards Corona Virus disease in north central Ethiopia, 2020.

A total of ten COVID 19 knowledge related question were asked. The mean (±SD) of knowledge score was 10.84 ± 1.24. Ninety five, (43.78%) had Good knowledge and one hundred twenty-two, 56.22 % had poor knowledge. Almost all, 98.16% reported that COVID-19 spread from person to person. Two third, 62% the participants respond as someone who has been released from COVID-19 quarantine is considered a risk for spreading the virus to others (Figure 3).

### 3.4 Preparedness of health care professionals

**Figure 4:**
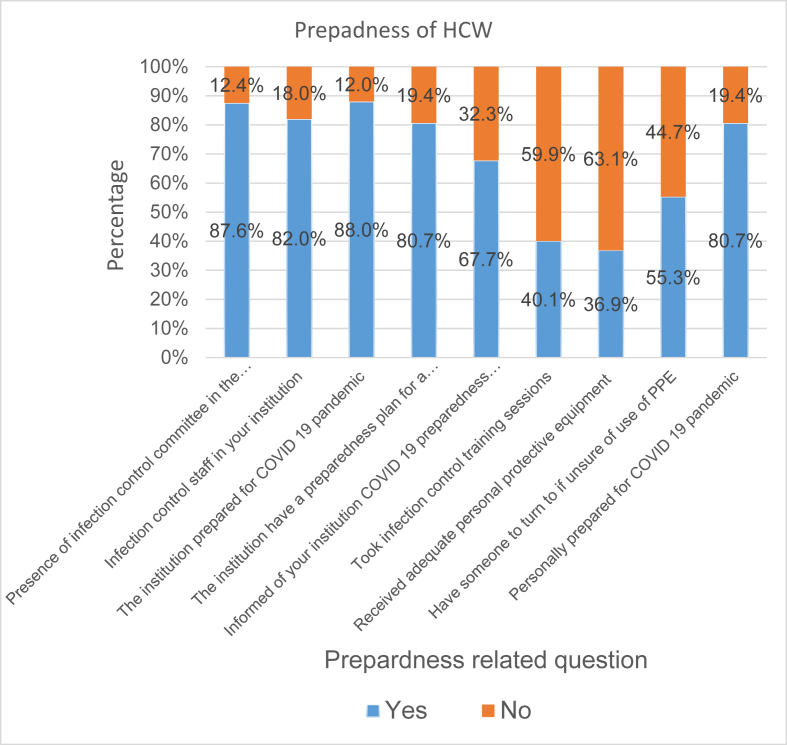
Preparedness of health care providers regarding COVID 19 pandemic north central Ethiopia.

The mean score for preparedness of HCWs was (11.79 ± 2.00). Half, 108 (49.77%) were ready for Corona virus pandemic. More than three fourth, 80.7% of HCWs were personally ready for tackling COVID 19. Similarly, 88.0% of HCWs reported as their institution is prepared for health care delivery system during the pandemic. Only 36.9% of HCWs received adequate personal protective equipment's (Figure 4). In addition, 44.57% reported fear of being infecting the general population, 30.86 % fear of infection themselves and the rest fear of infecting children.

### 3.5 Level of risk perception

A total of eleven questionnaires were used to assess the level of risk perception. The mean score (±SD) of risk perception was 16.35 ± 3.01. Majority, 125 (57.60%) have high risk perception of COVID 19.

### 3.6 Predictors of risk perception

Both bi-variable and multivariable logistic regression was undertaken. In binary logistic regression sex, marital status, knowledge, COVID 19 training, wear face mask outside, avoiding meeting a person suspected/confirmed of COVID 19 and commitment to health policy and procedures were significantly associated with level of risk perception. Whereas in multivariable analysis; knowledge, training and avoiding meeting a person suspected/confirmed of COVID 19 were statistically significant.

In this study, health care providers who had good knowledge related to corona virus were 2.83 times more likely to have high risk perception than HCWs with poor knowledge (AOR = 2.83; 95 CI:1.49, 5.34). HCWs having training related to COVID were 2.16 times more likely to have high risk perception of COVID 19 than HCWs who had no training (AOR = 2.16; 95 CI: 1.07, 4.39). In addition, those HCWs who avoid meeting a person suspected/confirmed of COVID 19 were 2.67 times more likely to have high risk perception towards corona virus than the counterparts (AOR = 2.67; 95 CI: 1.05, 6.83) (Table 2).

**Table 2:** Predictors of level of risk perception towards COVID 19 among health care providers working in north central Ethiopia,2020.

Variables	Risk perception		COR (Crude Odd Ratio)	AOR
	High n (%)	Low n (%)		
Sex				
Male	76 (35.02)	68 (31.34)	1	1
Female	49 (22.58)	24 (11.06)	1.83 (1.01.288)	1.50 (0.77,2.92)
Marital status				
Unmarried	48 (22.22)	48 (22.22)	1	
Married	77 (35.65)	43 (19.91)	1.79 (1.04,3.09)	1.21 (0.65,2.26)
Knowledge				
Good	69 (31.8)	26 (11.98)	3.13 (1.76, 5.56)	2.83 (1.49,5.34)∗
Poor	56 (25.8)	66 (30.41)	1	1
Training related to COVID -19				
Yes	105 (48.39)	60 (27.65)	2.8 (1.47,5.32)	2.16 (1.07,4.39)∗∗
No	20 (9.22)	32 (14.75)	1	1
Wear face mask				
Yes	114 (52.53)	74 (34.10)	2.52 (1.13,5.64)	1.89 (0.75,4.78)
No	11 (5.07)	19 (8.29)	1	1
Avoiding meeting a person suspected/confirmed of COVID 19				
Yes	114 (52.53)	73 (33.64)	2.7 (1.21, 5.99)	2.67 (1.05,6.83)∗∗∗
No	11 (5.07)	19 (8.76)	1	1
Less commitment to health policy and procedures				
Yes	71 (32.72)	39 (17.97)	1.79 (1.04,3.08)	1.80 (0.99,3.29)
No	54 (24.88)	53 (24.42)	1	1

∗, ∗∗, ∗∗∗ significant at P-value of: 0.001, 0.033, and 0.040 respectively.

## 4 Discussion

As COVID 19 pandemic is a highly contagious disease with high attack and case fatality rate, health care professional, community and institutional preparedness and risk perception towards the pandemic is important. The risk perception may have a negative impact on an individual and social well-being.

The study showed that overall preparedness was 49.8%. However, 80.7% of HCWs were personally ready for tackling COVID 19. This is consistent with the study conducted in Ghana 81.4% [32], china 77.17% [27]; higher than the study conducted in Singapore, 71.6% [36]. This could due to the difference in study period; the study in Singapore was conducted during influenza pandemic. Similarly, 80.7% of HCWs reported as their institution had preparedness plan for health care delivery system during the pandemic. This is lower than the study in Singapore,87.2% [36], but higher than Portuguese, 47.5% [37]. This could be due to difference in the study setting. In the current study only 36.9% of HCWs received adequate personal equipment's which affect overall preparedness of health care providers.

In current study, 57.6% (95% CI; 56.9, 58.3) had high risk perception on COVID 19. This is lower than the study conducted in Singapore during SARS, 76% [38], 68.3% in Ghana [32]. The possible difference could be the study in Singapore was during SARS epidemic which less worldwide attention than COVID 19 and 69.5% accepted the risk as their part of their Job. But, higher than the study conducted in Iran, 43.5% [39]. The rational could be the difference in target population, the current study is only health care professionals.

In this study, health care workers who had good knowledge related to corona virus were 2.83 times more likely to have high risk perception than HCWs with poor knowledge. This similar to the study conducted in Nigeria [40], china [41,42]. This could be as knowledge increase, ignorance towards the risk decrease [43], increase preventive behaviors [44] towards the disease. In addition, knowledge increase their sense of being infected, infection of their coworkers, family and the public.

HCWs having training related to COVID were 2.16 times more likely to have high risk perception of COVID 19 than HCWs who have no training. This is similar with the study conducted in Iran [45]. This could be due to training increase preventive behaviors of COVID 19 such as hand washing, social distancing, and increase adherence to preventive measures [46,47]. In addition, training increase the awareness of HCWs on consequences and change their attitude towards the pandemic.

In addition, those HCWs who did not meet a person suspected/confirmed of COVID 19 were 2.67 times more likely to have high risk perception towards corona virus than the counterparts. This is consistent to the study conducted in united kingdom [48], USA [49], and china [42]. The reason could be avoiding meeting a COVID 19 suspects/confirms is one preventive measure for corona virus transmission which in turn increase risk perception [45].

## 5 Conclusions

Risk perception, knowledge and preparedness was low. Knowledge, training on COVID 19 and avoiding meeting with suspected/confirmed COVID 19 client was significantly associated with risk perception. Ensuring the improvement of knowledge and preparedness by creating awareness, providing training and sharing the fate of other pandemic emerged before through different means of channels might be important in improving risk perception. In addition, ensuring psychological preparedness, increase supply of PPE, and encouragement is important. The government should focus on future plan on preparing health care professional for pandemics and outbreaks.

## Declarations

### Author contribution statement

Binyam Minuye Birihane:Conceived and designed the experiments; Performed the experiments; Analyzed and interpreted the data; Contributed reagents, materials, analysis tools or data; Wrote the paper.

Wubet Alebachew Bayih, Yohannes Tesfahun, Tigabu Munye, Abebaw Yeshambel Alemu, and Demeke Mesfin Belay: Analyzed and interpreted the data; Contributed reagents, materials, analysis tools or data; Wrote the paper.

### Funding statement

This research did not receive any specific grant from funding agencies in the public, commercial, or not-for-profit sectors.

### Data availability statement

Data included in article/supplementary material/referenced in article.

### Declaration of interests statement

The authors declare no conflict of interest.

### Additional information

No additional information is available for this paper.
